# The Enigmatic Odontogenic Keratocyst: A Cross-sectional Study of Odontogenic Cysts and Tumors Using Ki-67

**DOI:** 10.5041/RMMJ.10549

**Published:** 2025-07-31

**Authors:** Pretty Prince Panakkal, Pramod Philip Matthews, Soma Susan, Nivia Mahadoon, Pinky Varghese, Afshan Pallikkalakathu

**Affiliations:** 1 Department of Oral Pathology, Indira Gandhi Institute of Dental Sciences, Nellikuzhy, India; 2 Department of Oral Pathology, Mar Baselios Dental College, Kothamangalam, India; 3 Department of Oral Pathology, Government Dental College, Thiruvananthapuram, India; 4 Department of Prosthodontics, Indira Gandhi Institute of Dental Sciences, Nellikuzhy, India

**Keywords:** Ameloblastoma, dentigerous cyst, immunohistochemistry, Ki-67, odontogenic keratocyst, periapical cyst, proliferative marker

## Abstract

**Background:**

An odontogenic keratocyst is presently considered a cyst by the 2017 World Health Organization (WHO) classification, even though, at times, it shows highly aggressive behavior and a high recurrence rate. Ki-67 is a protein associated with the proliferative activity of the intrinsic cell populations. In tumors, Ki-67 is associated with tumor aggressiveness. This study aimed to compare the Ki-67 expression rates of odontogenic keratocysts to those of other odontogenic cysts and normal mucosa.

**Materials and Methods:**

A retrospective cross-sectional study was conducted using pathology samples retrieved from the archives of a tertiary care center to evaluate Ki-67 expression. Histopathologically confirmed cases of odontogenic keratocysts, radicular cysts, dentigerous cysts, and ameloblastomas were selected. The standardized immunohistochemistry streptavidin–biotin detection system HRP-DAB method was employed for analysis.

**Results:**

All the odontogenic keratocysts pathology samples demonstrated some degree of Ki-67 expression: mild, moderate, and severe Ki-67 expressions were identified in 26.7%, 53.3%, and 20.0% of the samples, respectively. Compared to the odontogenic keratocyst samples, the samples from dentigerous cysts, periapical cysts, ameloblastomas, and normal mucosa demonstrated no Ki-67 expression in 33.3%–66.7% of the samples, mild expression in 13.3%–40.0%, moderate expression in 0%–33.3%, and severe expression in none of the samples (*P*<0.001).

**Conclusions:**

Ki-67 was either moderately or severely overexpressed in the majority of odontogenic keratocyst samples. The 2017 WHO classification, which reclassifies keratocystic odontogenic tumors as cysts, conflicts with our findings.

## INTRODUCTION

Odontogenic cysts are the most common type of cyst occurring within the jaws, resulting from the proliferation and cystic degeneration of odontogenic epithelial rests.^1^ Odontogenic tumors are lesions derived from epithelial tissue, ectomesenchymal tissue, or a combination of both elements that have been part of the tooth-forming apparatus.^2^ The potential for further proliferation of these epithelial remnants leading to the formation of odontogenic cysts and tumors is different. It thus leads to variations in their molecular expression and biological behavior due to underlying mechanisms that remain highly speculative.^3^

Numerous papers focusing on the proliferative index of odontogenic keratocysts (OKCs) report that OKCs have a high mitotic index compared with the other types of odontogenic cysts, which gives rise to the hypothesis that OKCs represent benign cystic neoplasms instead of odontogenic cysts.^3^,^4^ In the 1971 and 1992 classifications of WHO, OKC was classified as a cyst. In 2005 the WHO reclassified OKC as a neoplasm and recommended the term keratocystic odontogenic tumor. The most controversial decision in the 2017 World Health Organization (WHO) classification was to reclassify keratocystic odontogenic tumors as cysts rather than neoplasms; however, at that time, evidence was lacking to justify otherwise.^5^ Compared to other odontogenic cysts, OKCs have a higher recurrence rate due to their fragile linings and tendency to form satellite cysts, hindering complete surgical removal. Incomplete enucleation, especially when the cyst is removed in fragments or when vital structures are preserved, further increases the chance of recurrence. Additional factors such as scalloped cyst margins and perforation of the lingual plate of the mandible have also been linked to a higher risk for recurrence. A new keratocyst developing adjacent to the site of the original lesion may sometimes be interpreted as a recurrence.^1^,^6^,^7^ Recurrence rates vary significantly depending on the site of involvement, with OKCs in the mandibular molar region having significantly higher recurrence rates than those in other locations.^8^ Furthermore, inactivation or mutation of the p53 tumor suppressor gene promotes uncontrollable proliferation, contributing to the aggressiveness and recurrence of OKCs.^1^

Malignant transformation of an OKC into primary intraosseous squamous cell carcinoma (PIOSCC) can occur—but rarely. Chronic inflammation is characterized by ongoing tissue damage and damage-induced cell proliferation. This persistent proliferation is often linked to dysplasia, a recognized precursor to carcinoma. Additionally, macrophages and T-lymphocytes in the inflammatory microenvironment can release tumor necrosis factor-alpha and macrophage migration inhibitory factor, further aggravating DNA damage. Ye et al.’s systematic review of malignant transformation of OKCs to PIOSCC identified 29 reported cases up to February 2020, covering studies from 2000 to 2019.^9^

Cell proliferation refers to cell growth and division. Markers of cell proliferation are most important for diagnostic and prognostic purposes.^1^ Most studies on cell proliferation evaluated antibodies such as Ki-67 and proliferating cell nuclear antigen (PCNA). Ki-67 is a nuclear protein with increased expression in the G1, S, G2, and M phases of the cell cycle.^2^ In contrast to PCNA, Ki-67 expression is a more reliable immunohistochemical tool for measuring proliferating activity as it is consistently absent in quiescent cells and is not detectable during DNA repair processes.^10^ In light of the controversy around the WHO 2017 reclassification of keratocystic odontogenic tumors as cysts, our study was aimed at evaluating and comparing Ki-67 expression in OKC, dentigerous cysts, radicular cysts, ameloblastomas, and clinically normal-appearing mucosa.

## METHODS

This retrospective cross-sectional study used pathology samples to evaluate Ki-67 expression. Samples were retrieved from the pathology department archives of the Mar Baselios Dental College, a tertiary medical center in Kerala, India. The institutional ethical committee authorized the study (IEC/17/ORALPATH/MBDC/2017 dated 20/11/ 2017).

We identified pathologic specimens diagnosed with OKC, dentigerous cyst, radicular cyst, ameloblastoma (follicular type), and clinically normal oral mucosa. The number of specimens evaluated per group was fifteen. Specimens were included if the patients from whom the specimens had been taken were otherwise healthy. Specimens from patients with systemic diseases or who were immunocompromised were excluded.

### Specimen Processing

All specimens were processed as follows: each sample was first deparaffinized by placing the slides on a warming table at 60°C for 1 h, followed by two sequential xylene baths for 10 min each. The slides were then rehydrated through a graded alcohol series (100%, 90%, and 70% alcohol) for 5 min each, followed by a 2-min rinse in wash buffer and a final rinse in distilled water. Antigen retrieval was done by immersing the slides in EDTA buffer and heating them in an induction cooker with pressure lid until pressure was reached (typically indicated by three whistles). The slides were then allowed to cool naturally to room temperature before further processing.

Immunohistochemical staining was performed using the streptavidin–biotin HRP-DAB detection system,^11^ at room temperature within a humidifying chamber. The procedure utilized the Peroxidase Detection System (Biogenex Life Sciences Pvt Ltd, Ambala, Haryana, India), which includes the following reagents: peroxidase block, protein/Power Block, primary antibody (anti-Ki-67 antigen), Super Enhancer, Super Sensitive label (secondary antibody), DAB-chromogen, DAB-substrate buffer, and hematoxylin. The primary antibody used was Ki-67 (clone MIB-1), a mouse monoclonal anti-human antibody (Biogenex Life Sciences Pvt Ltd).

The staining procedure began by removing excess buffer from the tissue sections through gentle tapping. Peroxidase block was then applied, and the slides were placed in a humidifying chamber for 10 min, followed by a 2-min rinse with wash buffer. Next, Power Block was applied after gently draining any remaining buffer, and the slides were returned to a humidifying chamber for an additional 10 min. Following buffer removal and wiping around the tissue sections, the primary antibody (diluted 1:100) was applied. The slides were incubated overnight in the humidifying chamber and then rinsed with Tris buffer for 2 min.

After removing excess buffer, the Super Enhancer reagent was applied and incubated for 30 min, followed by another 2-min rinse with Tris buffer. The Super Sensitive label, serving as the secondary antibody, was then applied for 30 min. Slides were again rinsed with Tris buffer for 2 min. This was followed by application of the substrate chromogen: the excess buffer was gently removed, and the slides were covered with DAB (3,3′-diaminobenzidine chromogen) and DAB buffer for 10 min, followed by a 5-min rinse in running water. Counterstaining was performed with a single dip in Harris hematoxylin, followed by gentle washing under running water.

Dehydration was performed using a graded alcohol series of 70%, 90%, and 100% for 5 min each, followed by two xylene baths for 10 min each. Finally, the slides were dried using filter paper and mounted using DPX (dibutyl phthalate in xylene), a non-aqueous permanent mounting medium, and cover slips.

### Determining the Ki-67 Labeling Index

The slides were examined microscopically, and cells were considered positive for Ki-67 antigen if any nuclear staining was observed. The expression of Ki-67 was assessed in five randomly selected, non-overlapping high-power fields at 40× magnification using manual light microscopy. The total labeling index was calculated using the following formula:


$$Total\;Labeling\;Index\;(\%)=\frac{\text{Total number of positive cells}\times 100}{\text{Total number of cells}}$$

To ensure antibody sensitivity, known positive controls—tonsil tissue and squamous cell carcinoma—were stained. Ki-67 expression was independently evaluated by two observers (Rater 1, the primary investigator; Rater 2, the co-investigator) according to the criteria shown in Table 1.^11^

**Table 1:** Scoring of Ki-67 Expression.

Score	% Cells with Positive Nuclear Staining
Nil	0%
Mild	<20%
Moderate	20%–40%
Severe	>40%

### Statistical Analysis

Numbers and percentages were used as summary statistics. Interobserver reliability was assessed using kappa statistics.^12^ The Ki-67 nil to moderate rates were compared to the moderate to severe expression rates between the different pathologies with the aid of the chi-square test. Data were analyzed using SPSS (SPSS Inc. Released 2007. SPSS for Windows, Version 16.0. Chicago, SPSS Inc.).

## RESULTS

Ki-67 expression in the different samples was scored by two independent observers (Table 2), and substantial interobserver reliability was observed (kappa value 0.78; *P*<0.001). Unlike in other cysts and in normal mucosa, Ki-67 was expressed in all OKCs (Table 3). Similarly, unlike in the other cysts and in normal mucosa, the degree of Ki-67 expression in the majority of OKCs was either moderate or severe. The tendency of OKCs to overexpress Ki-67 compared to the other histopathologies was significant (*P*<0.001).

**Table 2:** Ki-67 Expression Scores of Two Observers.

Count		Rater 2				Total
		Nil	Mild	Moderate	Severe	
**Rater 1**	**Nil**	33	1	0	0	34
	**Mild**	0	17	3	0	20
	**Moderate**	0	7	10	1	18
	**Severe**	0	0	2	1	3
**Total**		33	25	15	2	75

**Table 3:** Ki-67 Expression in the Study and Control Groups.

Ki-67 Expression	Odontogenic Keratocyst *n* (%)	Dentigerous Cyst *n* (%)	Periapical Cyst *n* (%)	Ameloblastoma *n* (%)	Normal Mucosa *n* (%)
Nil	0	10 (66.7)	10 (66.7)	5 (33.3)	9 (60)
Mild	4 (26.7)	3 (20)	2 (13.3)	5 (33.3)	6 (40)
Moderate	8 (53.3)	2 (13.3)	3 (20)	5 (33.3)	0
Severe	3 (20)	0	0	0	0
**All**	15 (100)	15 (100)	15 (100)	15 (100)	15 (100)

## DISCUSSION

In 2017, the WHO redefined OKC as a cyst rather than a neoplasm, citing insufficient evidence for designating it as a tumor. However, our findings, and those of previous studies, have demonstrated that OKC exhibits a significantly higher proliferative index, as measured by Ki-67 expression, than other odontogenic cysts and tumors. This observed biological behavior of OKCs forces the question: does the current classification fully reflect the unique characteristics of OKCs?

### Ki-67 and Proliferative Behavior of OKCs

According to de Vicente et al., Ki-67 expression strongly correlates with the mitotic index, making it a reliable indicator of proliferative activity.^3^ Ki-67 is a nuclear proliferative marker expressed during the active stages of the cell cycle. Its expression is consistently absent in quiescent cells (G0).

Our study demonstrated significantly higher Ki-67 expression in OKCs compared to other odontogenic cysts, ameloblastomas, and normal oral mucosa. Similar findings on Ki-67 overexpression in OKCs were reported by Modi et al., Brito-Mendoza et al., and Güler et al.,^8^,^13^,^14^ supporting the hypothesis that OKCs are intrinsically more proliferative than other odontogenic cysts and tumors. In contrast to OKCs, other odontogenic cysts and tumors generally exhibit lower and more variable Ki-67 expression patterns, reflecting differences in proliferative behavior and underlying growth mechanisms.^15^–^17^

Torres-Rendon et al. reported a Ki-67 positivity rate of 5.2% in normal oral epithelium, which is in line with our findings.^18^ In normal mucosa, Ki-67 staining was limited to occasional cells in the basal or parabasal layers, with no expression seen in the superficial layer.^19^ In our study, Ki-67 positivity in the basal layer may reflect normal physiological proliferation, as progenitor cells are located in this layer. Ki-67 is generally expressed, even at minimal levels, in normal epithelium. The absence of staining in some of our control samples may be due to technical factors, such as detection limits, rather than a true lack of proliferative activity.

In addition, the epithelial lining of OKCs in our study demonstrated prominent suprabasal Ki-67 positivity, consistent with findings by Wahba et al., who reported moderate nuclear staining in the suprabasal layer in 80% of OKCs.^20^ This pattern suggests increased proliferative potential in suprabasal cells—an atypical finding since mitogenic activity is usually predominant in the basal layer.

Beyond molecular markers, morphological changes observed in OKCs further support their proliferative potential. In our study, the epithelial lining of OKCs often showed pronounced infoldings into the capsule, a feature not commonly observed in other cyst types. Ahlfors et al. proposed that such infoldings may result from epithelial proliferation combined with collagenolytic activity and bone resorption.^21^

### Molecular and Cellular Evidence for Neoplastic Potential of OKCs

A previous study by Pan et al. revealed that higher OKC recurrence rates are associated with mutations in the *PTCH1* gene located on chromosome X. The OKC subgroups demonstrating *PTCH* mutation also had higher Ki-67 expression.^22^ This could be due to evasion of apoptosis and abnormal cell growth, as concluded by Barreto et al. in 2000.^23^ The *PTCH1* gene plays a critical role in the Sonic Hedgehog (SHH) signaling pathway by acting as a tumor suppressor that inhibits Smoothened (SMO). When *PTCH1* is mutated, this inhibition is lost, leading to the activation of GLI transcription factors and cell cycle genes, which in turn promote proliferation of odontogenic epithelium. 1

In our study, the increased Ki-67 expression observed in OKCs as compared to ameloblastoma and other odontogenic cysts suggests both increased proliferative activity and the involvement of molecular anti-apoptotic mechanisms. Hence, OKCs may therefore be considered to be a cystic neoplasm. Bcl-2, an anti-apoptotic protein that prolongs cell survival by inhibiting apoptosis, thereby promotes tumor development and treatment resistance. Co-expression of Bcl-2 and Ki-67 in the epithelial lining of OKCs may help explain their clinical aggressiveness and high recurrence rate.^24^

Several studies have also investigated the role of inflammation in OKC proliferation. Ayoub et al. suggested that Ki-67 expression in these lesions may be due to chronic inflammation.^16^ Similarly, Rodu et al. observed inflammation in 76% of OKCs, which was associated with a transition from a keratinized to a non-keratinized epithelial lining.^25^ Forssell et al. reported that OKCs with observable inflammation were more likely to recur.^26^ De Paula et al. further demonstrated significantly increased expression of PCNA, Ki-67, and AgNOR in inflamed OKCs compared to non-inflamed ones, concluding that inflammation may enhance epithelial proliferation by increasing the number of cycling cells and releasing cytokines and growth factors from the inflammatory infiltrate.^27^

The p53 tumor suppressor pathway has also been implicated in OKC biology. Slootweg et al. noted that p53 expression was concentrated in areas with high Ki-67 staining, indicating an association between p53 overexpression and increased proliferative activity in OKCs.^28^ The wild-type p53 protein functions as a transcription factor involved in growth arrest and apoptosis.^29^ Other studies have shown that p21 is a direct transcriptional target of p53, and that mutant forms of p53 fail to induce p21 expression. Ki-67 depletion has also been linked to reduced activity of the Dimerization partner, RB-like, E2F, and Multi-vulval class B (DREAM) complex, which depends on a functional p21 checkpoint.^30^–^32^ De Vicente et al. reported more p53-positive cells in OKC epithelium compared to dentigerous and radicular cysts, possibly reflecting overproduction or stabilization of wild-type p53 in response to increased proliferation.^3^ These findings suggest that OKCs may have impaired tumor suppressor function, contributing to their high proliferative potential—even in comparison to ameloblastomas.

Additional studies have shown increased expression of SHH-related proteins—SHH, Patched, and Smoothened—as well as Bcl-2 in OKCs, indicating resistance to apoptosis and offering a molecular explanation for their aggressive behavior and recurrence.^33^ Various *PTCH1* gene alterations have also been reported in OKCs, including missense mutations, frameshift mutations, and insertions that alter the amino acid sequence. Barreto et al. identified *PTCH1* mutations in odontogenic keratocysts—designated keratocystic odontogenic tumours (KCOTs) in the 2005 WHO classification but reverted to the cystic term OKC in the 2017 revision—including both lesions that arise in patients with nevoid basal cell carcinoma syndrome and truly sporadic cases; many of the variants are predicted to truncate the PTCH1 protein.^23^ These mutations likely disrupt normal Hedgehog signaling. Srinagesh et al. found higher *PTCH* gene expression in ameloblastomas than in OKCs. In addition, dysplastic changes have been observed in OKCs, and chronic inflammation has been associated with their transformation into squamous cell carcinoma.^34^–^36^

## LIMITATIONS AND FUTURE DIRECTIONS

This study was limited by its retrospective design and relatively small sample size. Further research with large cohorts and additional molecular markers is needed to advance the understanding of OKC pathogenesis and inform future updates to classification systems.

## CONCLUSION

The quantitative and qualitative differences in Ki-67 positivity among OKCs, dentigerous cysts, radicular cysts, and ameloblastoma suggest that OKCs exhibit increased Ki-67 expression, reflecting their intrinsic growth potential, recurrence, and aggressive behavior. These findings underscore the need to re-examine the 2017 WHO classification of OKCs in light of accumulating biological and molecular evidence. Nevertheless, while strong proliferative activity in OKCs is indicated by Ki-67 expression, this alone does not confirm a neoplastic nature. To determine whether OKCs should be classified as low-grade neoplasms rather than aggressive cystic lesions, further validation using genetic markers such as *PTCH1* mutations and p53 alterations is necessary.

## Abbreviations

OKC(s): odontogenic keratocyst(s)
PIOSCC: primary intraosseous squamous cell carcinoma
PCNA: proliferating cell nuclear antigen
WHO: World Health Organization.
